# Claudin-5 Affects Endothelial Autophagy in Response to Early Hypoxia

**DOI:** 10.3389/fphys.2021.737474

**Published:** 2021-08-31

**Authors:** Ping Yu, Yanyu Li, Gaoliang Zhong, Wen Li, Bing Chen, Jingjing Zhang

**Affiliations:** Affiliated Hospital of Guangdong Medical University & Key Laboratory of Zebrafish Model for Development and Disease of Guangdong Medical University, Zhanjiang, China

**Keywords:** Claudin-5, blood-brain barrier, autophagy, hypoxia, permeability

## Abstract

Hypoxic injury to cerebrovascular endothelial cells (ECs) after stroke leads to blood-brain barrier (BBB) dysfunction, which is commonly associated with disruptions of endothelial tight junctions (TJs) and increased permeability. Therefore, maintaining the structural integrity and proper function of the BBB is essential for the homeostasis and physiological function of the central nervous system (CNS). Our previous study revealed that autophagy functions on protecting the BBB by regulating the dynamics of Claudin-5, the essential TJ protein, under short-term starvation or hypoxia conditions. Here, we show that in zebrafish and *in vitro* cells, loss of membranous Claudin-5 conversely determine the occurrence of hypoxia-induced autophagy in cerebrovascular ECs. Absence of endothelial Claudin-5 could partly attenuate endothelial cell apoptosis caused by short-term hypoxic injury. Mechanism studies revealed that under hypoxic conditions, the existence of membranous Claudin-5 affects the stimulation of hypoxia inducible factor 1 subunit alpha (HIF-1a) and the inducible nitric oxide synthase (iNOS), which are responsible for the translocation of and endocytosis of caveole-packaged Claudin-5 into cytosol. Meanwhile, loss of Claudin-5 affects the generation of reactive oxygen species (ROS) and the downstream expression of BCL2/adenovirus E1B 19kDa protein interacting protein 3 (Bnip3). These together suppress the endothelial autophagy under hypoxia. This finding provides a theoretical basis for clarifying the mechanism of hypoxia-induced BBB injury and its potential protection mechanisms.

## Introduction

As a main part of the neurovascular units, the blood-brain barrier (BBB) is a physical barrier for the central nervous system (CNS) and is mainly composed of endothelial cells (ECs), tight junction (TJ) proteins, capillary basement membranes, pericytes, and astrocytes (Sweeney et al., 2019; Lochhead et al., 2020). The BBB exchanges nutrients and ions and tightly restricts toxic substances, inflammatory factors and immune cells in the peripheral blood from entering the CNS to maintain homeostasis. TJs are mainly composed of the proteins Claudin, Occludin, and Zonula Occludens-1 (ZO-1), which form the TJ structural skeleton that limits and regulates the permeability between cells and maintains barrier function (Wilhelm et al., 2016; Kadry et al., 2020). In the BBB, Claudin-5 is an important structural protein in the TJs connecting microvascular ECs, and it influences the development of cerebral blood vessels and the function of the BBB (Greene et al., 2019; Haruwaka et al., 2019; Hashimoto and Campbell, 2020). The clinicopathological characteristics of the BBB after ischemic stroke are evacuation of the TJ structure between ECs, a decrease in Claudin-5 protein expression, and an increase in permeability (Lv et al., 2018; Gholami et al., 2020). Therefore, to explore the mechanism by which Claudin-5 participates in the damage of BBB during cerebral ischemia has important clinical importance for the diagnosis and treatment of acute stroke, thrombolytic reperfusion, prognosis, and the prevention and treatment of hemorrhagic transformation after cerebral infarction.

Autophagy is a process by which cytoplasmic proteins or organelles are engulfed in vesicles, which fuse with lysosomes to form autophagic lysosomes; the contents of the lysosomes are degraded, thereby fulfilling the metabolic needs of the cell itself and the renewal of certain organelles. An increasing number of studies have shown that autophagy plays an important role in cerebral vascular pathology after stroke (Feng et al., 2017; Kim et al., 2018; Wang et al., 2018; Shi et al., 2021). Our studies used *in vitro* hypoxia or serum starvation models to mimic the onset of ischemic stroke, and we showed that early hypoxia/starvation could induce autophagy and the redistribution of membranous caveolin-1 (Cav-1) and Claudin-5 into cytosol, and autophagosomes mediate the degradation of cytosolically accumulated Claudin-5, thereby eliminating reactive oxygen species (ROS) and protecting the functional integrity of the endothelial barrier (Yang et al., 2019b, 2020).

In our subsequent study, we found that loss of membranous Claudin-5 in cerebrovascular endothelial cells could conversely inhibit the occurrence of autophagy in response to hypoxia. However, the molecular mechanism by which Claudin-5 affects endothelial autophagy is unclear. Here, by constructing both brain microvascular endothelial (bEnd.3) cell lines with stable *Claudin-5* knockout and zebrafish model with knockdown of *claudin-5b* by specific morpholino (MO), we revealed an underlying molecular mechanism by which Claudin-5 affects endothelial autophagy during the early period of hypoxic injury.

## Materials and Methods

### Cell Culture and Treatment

Mouse bEnd.3 were cultured in Dulbecco’s modified Eagle’s medium (DMEM, Gibco, C11995500BT) supplemented with 10% fetal bovine serum (FBS, Gibco) at 37°C in 5% CO_2._ The *Claudin-5^112Δ5^*-mutated bEnd.3 cell line was generated by the clustered regularly interspaced short palindromic repeats/CRISPR-associated protein 9 (CRISPR/Cas9) method using sgRNA (*ccaCAACATCGTGACGGCGCAGA*; Yang et al., 2021), and the *Claudin-5^297Δ4^*-mutated bEnd.3 cell line was generated by the CRISPR/Cas9 method using sgRNA (*cctTGACCGGCGCTCAGTGCACC*). The cells were stimulated with hypoxia as previously reported (Yang et al., 2019b, 2020). In brief, the cells were treated with 1% O_2_/94% N_2_/5% CO_2_. Then, the cells were incubated at 37°C in a constant temperature incubator for 2, 4, or 6h. Cell viability was examined by Cell Counting Kit-8 (CCK-8) assays.

### Establishment of bEnd.3 Cell Lines That Stably Express *RFP-GFP-LC3* Adenovirus

Endothelial bEnd.3 cells at a density of 80% were transfected with *RFP-GFP-LC3* adenovirus diluted in serum-free DMEM. Then, we changed the medium to DMEM with 10% FBS after 6–8h. After 3days, 2μg/ml puromycin was added to the adenovirus transfection and control wells. After 5days, monoclonal screening of transfected cells was performed.

### Measurements of Monolayer Endothelial Permeability

The trans-endothelial electrical resistance (TEER) and paracellular permeability were measured to reflect the barrier property of the endothelial monolayer as previously described (Yang et al., 2020). In brief, wild-type or *Claudin-5^112Δ5^*-mutated bEnd.3 cells were cultured on the trans-well plate of 0.4mm pore size (Millipore, United States) for a 5days. Then, the resistance of inserts was monitored by CellZscopeR-System (NanoAnalytics GmbH, Muenster, Germany). TEER value was calculated as Ω*cm^2^. For paracellular permeability measurement, the tightness of wild-type or *Claudin-5^112Δ5^*-mutated bEnd.3 monolayer cell was determined by the infiltration of FITC-labeled dextran (10kDa, 0.5mg/L, Thermo-Fisher, United States). After applying the dextran inside the well, the dye in the lower chamber was measured with a spectrophotometer-computer interfaced system (BioTek Epoch, United States) at a wave length of 594nm. For the measurements of TEER and permeation of tracers, three repeats of each measurement were investigated for each line.

### *Claudin-5* Rescue Assay in bEnd.3 Cells

*Claudin-5^112Δ5^*-mutated bEnd.3 cells at a density of 80% were transfected with *pIRES-eGFP* or *pIRES-eGFP-Claudin-5* according to the instructions of the Lipofectamine 2000 DNA transfection reagent (Invitrogen, 11668027). Then, we changed the medium to DMEM with 10% FBS after 6–8h. After 48h, the cells were stimulated with hypoxia for 4h, and the protein expression levels of Claudin-5, LC3 or inducible nitric oxide synthase (iNOS) were evaluated by immunostaining.

### Membrane and Cytosolic Protein Extraction Assay

Brain microvascular endothelial cells were cultured to form a confluent monolayer in a 10cm dish. Then the cells were exposed to hypoxic conditions for 4 and 6h, and we used a cell membrane and cytoplasm extraction kit (Beyotime, P0033). In brief, the cells were washed two times with cold PBS, and 1ml of membrane protein extraction reagent A was added. PMSF was added within a few minutes before use to a final concentration of 1mM. The cells were collected in a 1.5ml EP tube and placed on ice for 10–15min. Ultrasonic crushing was performed twice at 0.6Hz for 4s/time. The samples were centrifuged at 700*g* for 10min at 4°C, and the supernatant was collected and centrifuged at 14,000*g* for 30min at 4°C. The supernatant contained the cytoplasmic proteins, which were stored at −80°C. The supernatant contained 30–50μl of supernatant residue to avoid contact with the precipitate. The samples were centrifuged at 4°C and 14,000*g* for 10s. Then, 40μl of membrane protein extraction reagent B was added to the precipitate, vortexed for 5s, and placed in an ice bath for 5–10min, and the previous vortex and ice bath incubation steps were repeated 1–2 times. The samples were centrifuged at 14,000*g* for 5min at 4°C, and the supernatant was collected to obtain the cell membrane protein fraction.

### Immunofluorescence Assay

The cells were cultured to form a confluent monolayer in a 24-well plate. The cells were washed twice with cold PBS, and cold acetone was added and incubated for 10min. Then, the cells were washed once in cold 95% ethanol, once in cold 75% ethanol, and twice with PBS, after which the cells were blocked with blocking solution at room temperature for 1h (blocking solution: 1% BSA+0.5% Tween-20 in PBS). Then, the cells were incubated with the following primary antibodies at 4°C overnight (1:200, diluted with blocking solution): Claudin-5 (1:200; Invitrogen, 35–2,500), LC3A/B (1:200; Cell Signaling Technology, 4,108), Caveolin-1 (1:200; Santa Cruz Biotechnology, sc-894), hypoxia inducible factor 1 subunit alpha (HIF-1a; 1:200; ProteinTech, 20960-1-AP), and iNOS (1:100; ProteinTech, 18985-1-AP). The cells were then incubated with the following secondary antibodies at room temperature for 2h: Alexa Fluor 488-conjugated goat anti-mouse IgG (1:400; Thermo Fisher Scientific, A-11029) and Alexa Fluor 647-conjugated goat anti-rabbit IgG (1:400; Jackson ImmunoResearch, 111-605-003). DAPI (1:500; Sigma Aldrich, D9542) staining was also performed. For zebrafish tissue immunostaining, the brain sections were incubated with primary antibodies against Claudin-5 (Invitrogen, 35–2,500) and LC3B (Sigma-Aldrich, L7543), Alexa Fluor 647-conjugated goat anti-mouse IgG and Cy3-conjugated goat anti-rabbit IgG secondary antibodies and DAPI. Images were obtained with an Olympus FV3000 confocal laser microscope. Fluorescence quantitative analysis was performed using Image-Pro Plus software.

### Western Blotting

Western blotting was performed as described previously (Yu et al., 2019). In brief, protein samples were separated in 10–15% SDS-PAGE acrylamide gels, transferred onto PVDF membranes (Millipore, 0.22μm and blocked with 5% skim milk for 2h). The blots were then incubated at 4°C overnight with the following primary antibodies: Claudin-5 (1:1,000; Invitrogen, 34–1,600), LC3 (1:1,000; ProteinTech, 14600-1-AP), Caveolin-1 (1:200; Santa Cruz Biotechnology, sc-894), HIF-1a (1:1000; ProteinTech, 20960-1-AP), iNOS (1:1,000; ProteinTech, 18985-1-AP), LAMP-1 (1:1000; ProteinTech, 21997-1-AP), ATP1A1 (1:1,000, ProteinTech, 14418-1-AP), or β-actin (1:1,000; Servicebio, GB12001). Then, the blots were incubated at room temperature for 2h with HRP conjugated anti-rabbit or anti-mouse IgG (H+L; 1:1,000; Servicebio) secondary antibodies. Western blot bands were analyzed by adding ECL advance Western blotting detection reagents (Thermo, 34,580) and imaged using a SmartChemi-500 imaging system (Sage Creation Science, China). The immunoblot bands were quantitatively analyzed by ImageJ software.

### Flow Cytometry Analysis

Intracellular ROS were measured using 2',7'-dichlorofluorescein diacetate (DCFH-DA, Beyotime, S0033S). DCFH-DA was diluted with serum-free medium to a final concentration of 10μM. The cells were harvested in a 15ml centrifuge tube, and 1ml of diluted DCFH-DA was added to the cells and incubated for 20min at 37°C. The cells were washed three times with serum-free DMEM. Then, detection was performed by flow cytometry. Cell apoptosis was detected by Annexin V-FITC cell apoptosis detection kit (Beyotime, C1062M). Briefly, cells were harvested and stained with annexin V-FITC and propidium iodide for 20min at room temperature and examined using a flow cytometer.

### Statistical Analysis

For statistical analysis, GraphPad Prism 5 software was used. All values are presented as the means±SD, and two-tailed Student’s *t*-test or one-way ANOVA was applied to determine statistical significance (^*^p<0.05, ^**^p<0.01 and ^***^p<0.005).

## Results

### Involvement of Endothelial Claudin-5 in Autophagy Under Hypoxic Condition

Our previous study has shown the activation of autophagy in endothelial cells in response to hypoxia (Yang et al., 2019b, 2020). To confirm the alterations of autophagic flux in bEnd.3 cells in response to hypoxia, bEnd.3 cells were transfected with *RFP-GFP-LC3* adenovirus for live imaging analysis. As shown in Supplementary Figure S1, hypoxia induction increased the numbers of both GFP and RFP dots in bEnd.3 cells compared with that in normoxia-treated cells, and we found that the red/yellow dot ratio increased due to hypoxia induction, indicating an increased expression of LC3, a marker of autophagy, and a formation of autophagosomes to autolysosomes in hypoxia-treated cells. Previous studies have shown that short-term hypoxia induction causes the redistribution and endocytosis of membranous Claudin-5, and autophagy is then activated to mediate the degradation of abnormally accumulated Claudin-5 in cytosol, thereby eliminating ROS and protecting the integrity of the BBB barrier from fast injury (Yang et al., 2019b, 2020).

### The Impact of Claudin-5 on the Activation of Autophagy Under Early Hypoxia Induction

To explore the impact of the membranous TJ protein Claudin-5 on the occurrence of autophagy in response to hypoxia, two stabilized *Claudin-5*-knocked out bEnd.3 mutated cell lines, *Claudin-5^112Δ5^* with a 5bp deletion and *Claudin-5^297Δ4^* with a 4bp deletion, were first constructed and generated by CRISPR/Cas9 strategy (Supplementary Figure S2A). The expression of Claudin-5 was examined by immunofluorescence staining and immunoblotting respectively, and the results indicated a total loss of Claudin-5 from cell membrane in these lines (Supplementary Figures S2B,C). To verify the endothelial barrier function of bEnd.3 cells after loss of Claudin-5, the TEER and paracellular permeability of the wild-type or *Claudin-5^112Δ5^* mutated bEnd.3 monolayer cells were measured. The results indicated a significant loss of the tightness of monolayer *Claudin-5^112Δ5^* mutated cells in comparison to that of the wild-type bEnd.3 cells (Supplementary Figures S2D,C). Since a functional alteration was observed in bEnd.3 cells after loss of Claudin-5, we additionally analyzed the cell viability in response to hypoxia by CCK-8 assays in both wild-type and mutated bEnd.3 cells. The result showed that the survival rate of both *Claudin-5*-knocked out cells was still above 90% till 6h post-hypoxia induction, and there was no significant variations of the cell viability among the wild-type and *Claudin-5*-knocked out lines under short term hypoxic induction (Supplementary Figure S3).

**Figure 1:**
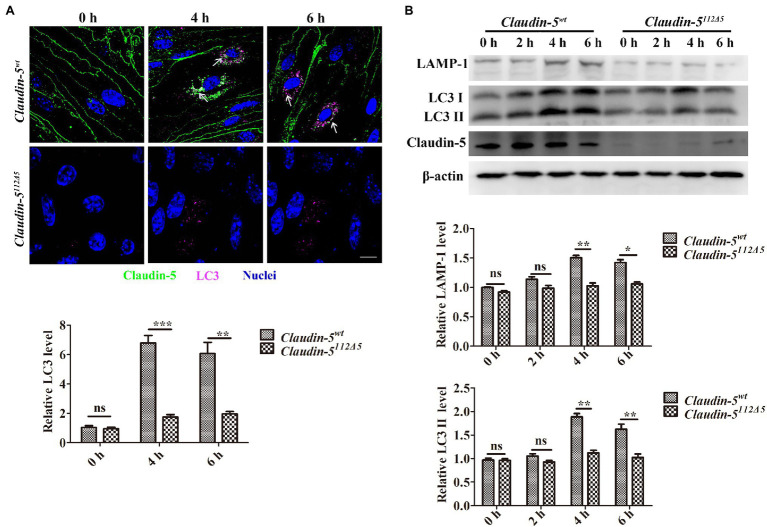
The specificity of the involvement of Claudin-5 in autophagy under hypoxia induction. **(A)** Confocal microscopy images of Claudin-5 (green) and LC3 (purple). Colocalization of Claudin-5 with LC3 (white arrow) and quantitative analysis of LC3 levels by Image-Pro Plus. Mean±SD, *n*=3 independent experiments per group. Scale bar, 10μm. **(B)** Western blot analysis of the autophagy markers LC3 and LAMP-1. Mean±SD, *n*=3 independent experiments per group. ^*^p<0.05 ^**^p<0.01 and ^***^p<0.005.

Next, to verify whether Claudin-5 affects the occurrence of autophagy in response to hypoxia, the expression of LC3 in *Claudin-5^wt^* and *Claudin-5^112Δ5^* bEnd.3 cells were analyzed by immunofluorescence staining first. As a result, it was found that in bEnd.3 *Claudin-5^wt^* cells, Claudin-5 and LC3 showed obvious colocalization in the cytoplasm after 4 or 6h hypoxia induction (white arrows in Figure 1A). The expression level of LC3 in cells with *Claudin-5* knockout was significantly reduced (Figure 1A). Meanwhile, the expression levels of LC3 and LAMP-1, a lysosomal membrane protein and a specific marker of autolysosomes (Zhan et al., 2018), in *Claudin-5^wt^* and *Claudin-5^112Δ5^* bEnd.3 cells in response to hypoxia were analyzed by immunoblotting. The protein expression levels of both LC3 and LAMP-1 in bEnd.3 *Claudin-5^112Δ5^* cells were significantly lower than those in *Claudin-5^wt^* bEnd.3 cells after 4 or 6h hypoxia induction (Figure 1B). The inhibition of the autophagy activation was confirmed by another *Claudin-5* mutant bEnd.3 line of *Claudin-5^297Δ4^*, where the expression of both LC3 and LAMP-1 was sufficiently reduced due to the loss of Claudin-5 in response to hypoxia induction for 4 or 6h (Supplementary Figure S4). A rescue experiment was also performed to further verify the specificity of Claudin-5 on hypoxia-induced autophagy occurrence. The *pIRES-eGFP* or *pIRES-eGFP-Claudin-5* plasmid was first transfected into mutated *Claudin-5^112Δ5^* bEnd.3 cells. The followed immunofluorescence staining showed that cells successfully transfected with the *pIRES-eGFP-Claudin-5* plasmid expressed LC3 at levels higher than the vehicle in response to 4h hypoxia induction (Supplementary Figure S5). These results revealed that endothelial membranous Claudin-5 was specifically involved in the activation of autophagy during the early stage of hypoxia.

**Figure 2:**
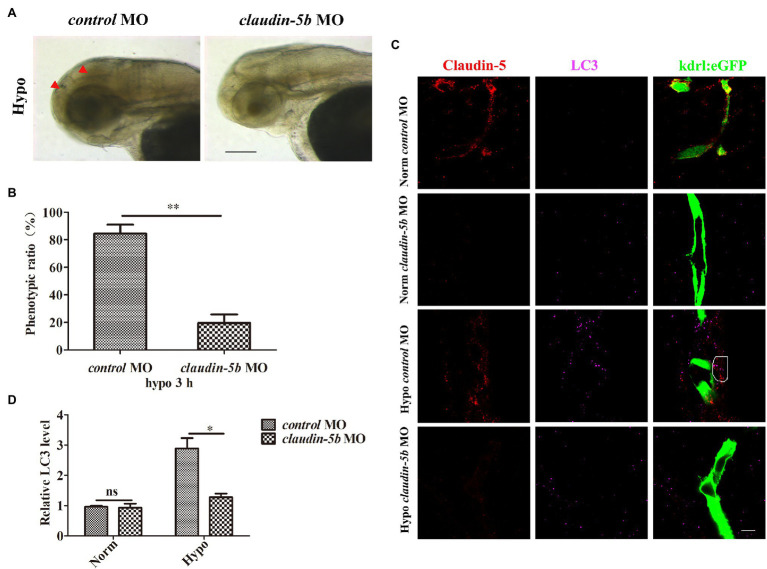
Loss of Claudin-5b in zebrafish embryos inhibits activation of autophagy in cerebrovascular endothelial cells (ECs). Morpholino (MO) silencing of *claudin-5b* in endothelial eGFP-specific transgenic *Tg*(*kdrl:eGFP*) zebrafish embryos. **(A)** Brain phenotype (red arrowheads) of *control* MO and *claudin-5b* MO zebrafish larvae in response to hypoxia. Scale bar, 100μm. **(B)** Quantitative analysis of the severe injury rates of *control* MO and *claudin-5b* MO zebrafish larval brains. Mean±SD, *n*=3 independent experiments per group. **(C)** Confocal microscopy images of Claudin-5 (red) and LC3 (purple). Scale bar, 5μm. **(D)** Quantitative analysis of LC3 levels by Image-Pro Plus. Mean±SD, *n*=3 independent experiments per group. ^*^p<0.05 and ^**^p<0.01.

We also sought *in vivo* evidences supporting the correlations between Claudin-5 and autophagy activation in endothelial cells under hypoxia. To this end, endothelial eGFP-specific transgenic *Tg(kdrl:eGFP)* zebrafish was applied to investigate the autophgy activation in endothelial cells (Yang et al., 2019b, 2020). Since zebrafish Claudin-5b is the main Claudin expressed in cerebrovascular endothelial cells during the embryonic stages (Yang et al., 2021), MO-mediated silencing of *claudin-5b* in zebrafish embryos was first performed. After 3days, it was found that in response to 3h hypoxia treatment, the control morphant embryo presented much severer brain injury than that of the *claudin-5b* morphants (red arrowheads, Figures 2A,B). Immunofluorescence staining with the embryonic brains further revealed that in control morphant zebrafish larvae endothelial cells, the expression level of LC3 was higher than that in the cerebrovascluar endothelial cells of *claudin-5b* morphants post-hypoxia induction (Figures 2C,D). Above *in vivo* and *in vivo* evidences confirmed that loss of Claudin-5 could partly affect the activation of endothelial autophagy caused by hypoxia induction.

Since, we have observed a higher phenotype ratio of brain injury in control embryos in comparison to that in the brain of *claudin-5b* morphants post-hypoxia treatment, we wondered whether lack of endothelial Claudin-5 could alleviate the cell injury or death caused by hypoxia in a certain extent. Therefore, we analyzed hypoxia-caused apoptosis of the *Claudin-5^wt^* and *Claudin-5* knocked out bEnd.3 cells. The results indicated that, after 4h-hypoxia induction, *Claudin-5^wt^* bEnd.3 cell showed a higher apoptosis ratio than *Claudin-5^112Δ5^* or *Claudin-5^297Δ4^* bEnd.3 cells (Supplementary Figure S6).

### Claudin-5 Affects Caveolae-Mediated Endocytosis Under Hypoxia Conditions

**Figure 3:**
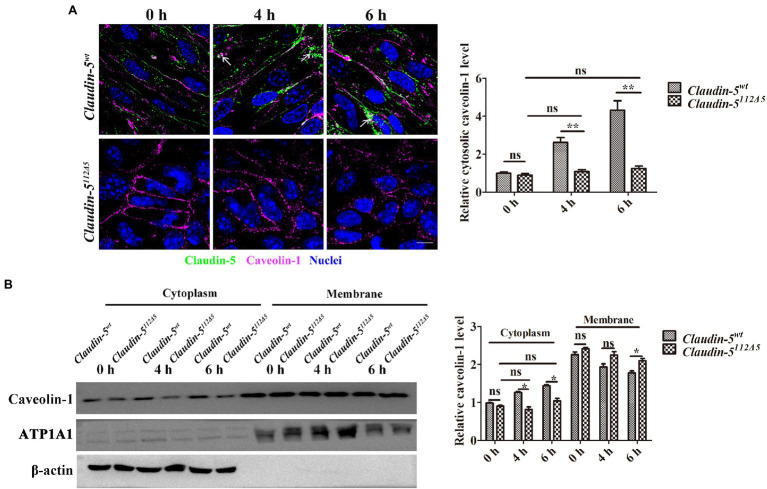
Caveolin-1 (Cav-1)-mediated Claudin-5 redistribution in response to hypoxia. **(A)** Confocal microscopy images of Claudin-5 (green) and Cav-1 (purple). Colocalization of Claudin-5 and Cav-1 (white arrows). Mean±SD, *n*=3 independent experiments per group. Scale bar, 10μm. **(B)** Western blot analysis of Cav-1 in the cytoplasm and membrane. Mean±SD, *n*=3 independent experiments per group. ^*^p<0.05 and ^**^p<0.01.

It has been reported that in response to hypoxia induction, membranous Claudin-5 in the vascular endothelial cells is packaged by Cav-1-composed caveolae and is then endocytosed into the cytoplasm, where it is eventually degraded by autophagosomes or autolysosomes (Liu et al., 2012, 2016). Therefore, we next asked whether the expression and localization of Cav-1 in hypoxia-treated bEnd.3 cells will be altered since Claudin-5 is absent. To this end, an immunofluorescence staining against Cav-1 was first performed in *Claudin-5^wt^* and *Claudin-5^112Δ5^* bEnd.3 cells after 4 or 6h hypoxia induction. The results indicated that most of the Cav-1 still localized in the membrane of *Claudin-5^112Δ5^* bEnd.3 cells due to the loss of Claudin-5, and that the Cav-1, which colocalized with Claudin-5 in the cytosol of *Claudin-5^wt^* bEnd.3 cells was much more than that in *Claudin-5^112Δ5^* bEnd.3 cells in response to hypoxia for 4 and 6h (Figure 3A). There was no significant effect of accumulation or endocytosis of Cav-1 in bEnd.3 *Claudin-5^112Δ5^* cells in response to hypoxia for 4 and 6h (Figure 3A). The protein level of Cav-1 in the cell membrane and cytoplasm was also quantified by immunoblotting analyses. The results confirmed that due to lack of Claudin-5, there was almost no loss of membranous Cav-1 post-hypoxia induction for 6h, and there was no significant accumulation of Cav-1 in the cytoplasm either in comparison with that of the *Claudin-5^wt^* bEnd.3 cells (Figure 3B). These results confirmed that Cav-1 is mainly responsible for the endocytosis of membranous Claudin-5 in response to hypoxia induction, while in the absence of Claudin-5, the delocalization of Cav-1 from endothelial membrane is sufficiently inhibited.

### The Effects of Endothelial Claudin-5 on the Expression of iNOS and HIF-1a Under Hypoxia

**Figure 4:**
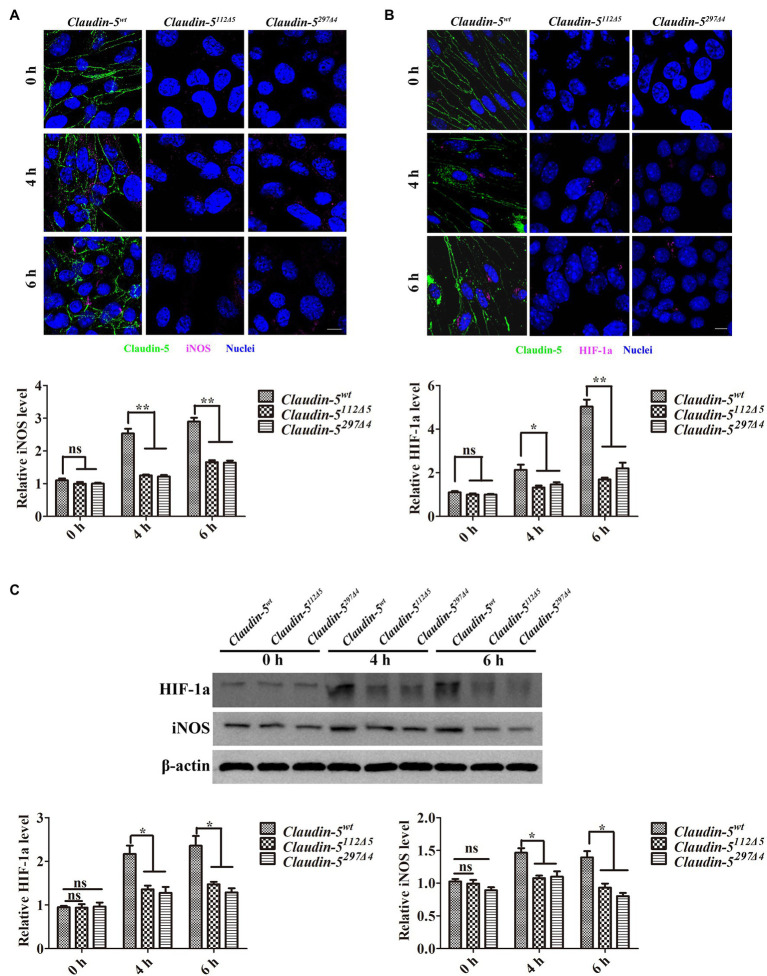
Inducible nitric oxide synthase (iNOS) induces Cav-1-mediated Claudin-5 translocation in response to hypoxia. **(A,B)** Confocal microscopy images of Claudin-5 (green), iNOS (purple), and hypoxia inducible factor 1 subunit alpha (HIF-1a; purple). Scale bar, 10μm. Mean±SD, *n*=3 independent experiments per group. **(C)** Western blot analysis of iNOS and HIF-1a. Mean±SD, *n*=3 independent experiments per group. ^*^p<0.05 and ^**^p<0.01.

Previous studies have shown that iNOS-derived NO is the main cause of Cav-1-mediated Claudin-5 ectopic activity in response to hypoxia (Liu et al., 2016; Yang et al., 2019a). HIF-1a is a transcriptional regulator produced by the body during hypoxia, and HIF-1a is one of the main factors that regulates iNOS transcription (Robinson et al., 2011). Therefore, to ask whether the loss of Claudin-5 could conversely affect the production of iNOS and its upstream regulator of HIF-1a, we next analyzed the their expression levels in both wild-type and *Claudin-5* mutant bEnd.3 cells after 4 or 6h hypoxia induction by immunofluorescence staining and immunoblotting. We found that the expressions of both iNOS and HIF-1a in *Claudin-5* mutant bEnd.3 cells were significantly lower than that in *Claudin-5^wt^* cells in response to hypoxia induction for 4 and 6h (Figures 4A,B). To confirm the specificity of membranous Claudin-5 on endothelial iNOS generation under hypoxia, rescue experiments by transfecting *pIRES-eGFP-Claudin-5* plasmid in *Claudin-5* mutant bEnd.3 cells were performed. It was found that restored expression of Claudin-5 in bEnd.3 *Claudin-5^112Δ5^* mutant cells could recover the generation of iNOS in response to hypoxia treatment for 4h (Supplementary Figure S7). Immunoblotting analyses also revealed that the protein expression levels of HIF-1a and iNOS in wild-type bEnd.3 cells after 4 and 6h of hypoxia were higher than that in *Claudin-5^112Δ5^* and *Claudin-5^297Δ4^* bEnd.3 cells (Figure 4C), indicating that lack of membranous Claudin-5 could partly inhibited the production of endothelial HIF-1a and iNOS under short term hypoxia induction.

### ROS Generation Was Affected by Claudin-5 in bEnd.3 Cells Post-hypoxia Induction

**Figure 5:**
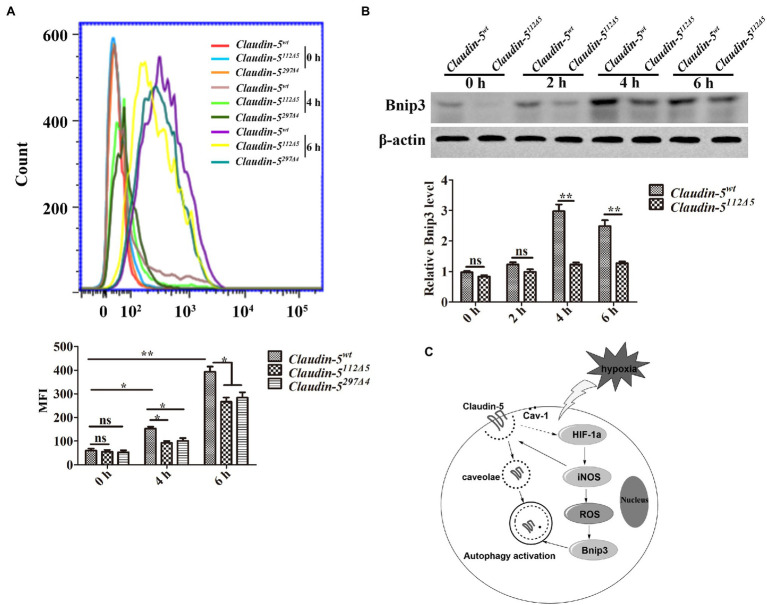
Reactive oxygen species (ROS) production drives BCL2/adenovirus E1B 19kDa protein interacting protein 3 (Bnip3) expression in brain microvascular endothelial (bEnd.3) cells in response to hypoxia. **(A)** ROS analysis by flow cytometry and quantitative analysis of ROS intensity. **(B)** Western blot analysis of Bnip3. Mean±SD, *n*=3 independent experiments per group. ^*^p<0.05 and ^**^p<0.01. **(C)** Schematic illustrating the mechanism that Claudin-5 is involved in autophagy in cerebral vascular endothelial cells in the early stage of hypoxia.

ROS are produced by various sources in the CNS, including mitochondria, NADPH oxidase, and NOS, especially under hypoxia conditions (Yamamoto et al., 2006; Kuthati et al., 2019). We used DCFH-DA to measure intracellular ROS and then analyzed these levels by flow cytometry. It was found that the ROS level in *Claudin-5^wt^* bEnd.3 cells was higher than those in both *Claudin-5^112Δ5^* and *Claudin-5^297Δ4^* mutant bEnd.3 cells in response to hypoxia induction for 4 and 6h, respectively (Figure 5A). This also suggests that the production of cellular ROS may be directly related to NOS in response to hypoxia. Previous studies have shown that under hypoxia induction, ROS could function on driving the expression of Bnip3 (Bcl-2 and adenovirus E1B 19-kDa interacting protein 3), which is a single transmembrane protein mediating autophagy to maintain cell survival (Zhang et al., 2019). Hence, immunoblotting analysis was performed to detect the protein expression level of Bnip3 in *Claudin-5* mutated bEnd.3 cells. The results revealed that in comparison with the expression in wild-type control bEnd.3 cells, the expression level of Bnip3 in *Claudin-5^112Δ5^* bEnd.3 cells was significantly lower after 4 or 6h hypoxia induction (Figure 5B). Above results indicated that the existence of membranous Claudin-5 affects the production of ROS and the downstream Bnip3 expression in response to early hypoxia induction.

In summary, based on our previous finding that under early hypoxia injury, autophagy protects the BBB from fast breakdown by regulating the dynamics of Claudin-5, we further revealed the existence of membranous Claudin-5 could conversely affect the occurrence of autophagy in endothelial cells by both *in vivo* and *in vitro* evidences (Figure 5C). This is probably due to the attenuated endocytosis of caveolae-packaged Claudin-5 and reduced autophagic degradation of abnormally accumulated proteins in cytosol during the early hypoxia induction stages (as early as 6h). Meanwhile, our results indicate that lack of endothelial Claudin-5 caused a decreased sensitivity and production of HIF-1a and iNOS to early hypoxia induction, which together with the downstream ROS and Bnip3, affect the endothelial autophagy activation.

## Discussion

Our previous have shown that under early stage of starvation/hypoxia induction, endothelial membranous Claudin-5 was endocytosed from the cell membrane into the cytoplasm (Yang et al., 2019b, 2020). Additionally, we also revealed that endothelial autophagy functions on the clear of the abnormally accumulated cytosolic Claudin-5 and caveolin-1, therefore, protects endothelial cell from apoptosis and maintains the integrity of the endothelial barrier (Yang et al., 2019b, 2020). In this study, it is found that the activation of autophagy was partly inhibited in *Claudin-5* knocked out bEnd.3 cells, indicating membranous TJ protein of Claudin-5 could conversely affect the endothelial autophagy under hypoxia conditions. Therefore, two questions were raised regarding (1) the specific involvement of Claudin-5 in autophagy activation and (2) the mechanism by which Claudin-5 affects endothelial autophagy in the early stage of hypoxia treatment.

To identify the correlations between Claudin-5 and autophagy activation, two stable *Claudin-5* knockout mutant cell lines (bEnd.3 *Claudin-5^112Δ5^* and bEnd.3 *Claudin-5^297Δ4^*) were successfully constructed, respectively, by CRISPR/Cas9 strategy in this study. Meanwhile, the endothelial *claudin-5b* knocked down zebrafish embryo was used as *in vivo* model to identify the activation of autophagy under hypoxia conditions. Both *in vivo* and *in vitro* evidences confirmed the findings that the membrane expression of Claudin-5 affects the occurrence of endothelial autophagy in response to short-term hypoxia induction. We further explored the underlying mechanism by which Claudin-5 is involved in autophagy activation during early stage of hypoxia treatment. We have shown that cerebral ischemia can cause Cav-1-mediated redistribution and endocytosis of Claudin-5, which causes BBB disruption in the early stages of stroke (Yang et al., 2020). Meanwhile, previous studies have shown that knock down of *Cav-1* completely eliminates the redistribution of Claudin-5 induced by hypoxia and partially prevents the destruction of the BBB (Liu et al., 2016), indicating a tight correlation of membranous Cav-1 and Claudin-5, and a necessity of Cav-1 in membranous Claudin-5 endocytosis. Combining our findings with reduced protein expressions of cytoplasmic Cav-1 in bEnd.3 *Claudin-5^112Δ5^* cells compared with that in bEnd.3 *Claudin-5^wt^* cells under hypoxia, we conclude that absent of membranous/endocytosed Claudin-5 and reduced Cav-1 in cytosol suppresses the activation of endothelial autophagy under hypoxia induction due to the lack of cytosolic substrates for the autophagic degradation (Figure 5C).

Liu et al. (2016) has reported that nitric oxide (NO) produced by iNOS could also induce Cav-1-mediated Claudin-5 redistribution in response to hypoxia. NOS has three types: neuronal (nNOS or NOS1), endothelial (eNOS or NOS3), and inducible (iNOS or NOS2). These NOS subtypes increase after cerebral ischemia. iNOS is different from eNOS and nNOS, and iNOS produces a large amount of NO (μM to mM range) in response to various stimuli such as LPS, cytokines, or hypoxia/ischemia (Kirk et al., 1990; Lapointe et al., 2006; Lakhan et al., 2009; Costa et al., 2016). In this study, the iNOS expression level in *Claudin-5^112Δ5^* and *Claudin-5^297Δ4^* mutant bEnd.3 cells was much lower than that in *Claudin-5^wt^* bEnd.3 cells in response to hypoxia as shown by immunofluorescence staining and immunoblotting analyses. HIF-1a, as a transcriptional regulator produced most of cells during hypoxic injury, is one of the main regulators of iNOS transcription (Nizet and Johnson, 2009; Takeda et al., 2010). Our data showed that after short period hypoxia induction, the expression level of HIF-1a in *Claudin-5^112Δ5^* and *Claudin-5^297Δ4^* mutant bEnd.3 cells was significantly lower than that in *Claudin-5^wt^* bEnd.3 cells. As a downstream effecter of iNOS, ROS is usually produced by various sources in the CNS, including mitochondria, NADPH oxidase, and NOS (Galley, 2011; Chan and Chan, 2014; Steinz et al., 2020). We used DCFH-DA to measure the ROS level in endothelial cells, and the data showed that the ROS generation in *Claudin-5^wt^* bEnd.3 cells were higher than those in *Claudin-5^112Δ5^* and *Claudin-5^297Δ4^* bEnd.3 cells in response to early hypoxia stimulation. Previous studies have shown that ROS could drive the expression of downstream Bnip3 and promote the occurrence of autophagy in response to hypoxia (Xu et al., 2015; Zhang et al., 2019). Additionally, we analyzed the Bnip3 expression in *Claudin-5^112Δ5^* bEnd.3 cells, and the immunoblotting analyses showed that loss of Claudin-5 efficiently inhibited Bnip3 expression in hypoxia-treated endothelial cells. This probably also suppresses the activation of autophagy to a certain degree. We speculate that the existence of membranous Claudin-5 affects the HIF-1a/iNOS/ROS/Bnip3 pathway might be due to a reduced sensitivity of *Claudin-5*-mutated endothelial cells to the short term hypoxia stimulation. On the other hand, we have reported in our previous studies that autophagy is responsible for the degradation of cytosolic Claudin-5 induced by early hypoxia treatment (shorter than 12h), while only under long-period hypoxia induction (24h), TJ protein of ZO-1 is selectively degraded by autophagy (Yang et al., 2020). Besides, the degradation of another TJ protein of Occludin is depending on the ubiquitin/proteasome system instead of autophagic pathway (Traweger et al., 2002). Therefore, it is possible that autophagy is mainly responsible for the specific degradation of endocytosed Claudin-5, and the repression of endothelial autophagy during early hypoxia induction is due to lack of delocalized cytosolic Claudin-5 to degrade.

As an important membranous TJ protein, endothelial Claudin-5 functions on maintain the integrity and tightness of endothelial barriers. Lack of Claudin-5 in cerebral endothelial cells causes a leakage of BBB (Nitta et al., 2003). Here, although mutation/loss of Claudin-5 in bEnd.3 cells caused a decrease of monolayer endothelial cell barrier function, it showed no affect on the cell viability even under the hypoxia conditions for longer than 6h. Moreover, it is surprising to find in our study that under early hypoxia induction, absence of membranous Claudin-5 could increase the resistance of endothelial cell apoptosis to the hypoxic injury, irrespective of the loss of its endothelial barrier functions. The hypoxia treatment assay on zebrafish embryos confirmed that knockdown of endothelial *claudin-5* in the vascular endothelial cells is beneficial to the survival ratio of CNS cells. This might be because in the endothelial cells missing membranous Claudin-5, there is weak cytotoxicity due to decreased accumulations of abnormal proteins in cytosol, which helps with the cell survive from the hypoxic injury.

In summary, this study reveals a previously unknown but essential function of membranous Claudin-5 on activating autophagy in cerebrovascular endothelial cells during early stage of hypoxia induction. Combing with the previous reports that autophagy is able to mediate the degradation of cytosolic Claudin-5 to protect endothelial barrier from fast injury under hypoxia treatment, our findings reveal a bidirectional regulatory mechanisms of TJ protein Claudin-5 and endothelial autophagy under hypoxic conditions. These studies may provide theoretical basis for clarifying the mechanism of BBB injury and also potential emergency protection mechanisms in stroke.

## Data Availability Statement

The original contributions presented in the study are included in the article/Supplementary Material, further inquiries can be directed to the corresponding author.

## Ethics Statement

The animal study was reviewed and approved by Guangdong Medical University. Handling of zebrafish was performed in accordance with Guangdong State Regulations on Laboratory Animal Management.

## Author Contributions

PY performed the experiments, analyzed the data, and wrote the manuscript. YL and GZ generated the mutated cell lines and performed the *in vivo* experiments. WL and BC gave extract suggestions on this study. JZ initiated the study, designed the experiments, analyzed the data, and wrote the manuscript. All authors contributed to the article and approved the submitted version.

## Conflict of Interest

The authors declare that the research was conducted in the absence of any commercial or financial relationships that could be construed as a potential conflict of interest.

## Publisher’s Note

All claims expressed in this article are solely those of the authors and do not necessarily represent those of their affiliated organizations, or those of the publisher, the editors and the reviewers. Any product that may be evaluated in this article, or claim that may be made by its manufacturer, is not guaranteed or endorsed by the publisher.

## Abbreviations

BBB: Blood-brain barrier
CNS: Central nervous system
bEnd.3 cells: Brain microvascular endothelial cells
HIF-1a: Hypoxia inducible factor 1 subunit alpha
iNOS: Inducible nitric oxide synthase
Cav-1: Caveolin-1
ROS: Reactive oxygen species
ECs: Endothelial cells
TJ: Tight junction
ZO-1: Zonula occludens-1
MO: Morpholino
CRISPR/Cas9: Clustered regularly interspaced short palindromic repeats/CRISPR-associated protein 9
DCFH-DA: 2',7'-dichlorofluorescein diacetate
DMEM: Dulbecco’s modified Eagle’s medium
FBS: Fetal bovine serum
Bnip3: BCL2/adenovirus E1B 19kDa protein interacting protein 3
MFI: Mean fluorescence intensity
TEER: Trans-endothelial electrical resistance
