# Supplementary material for: Claudin-5 Affects Endothelial Autophagy in Response to Early Hypoxia
Source: Front Physiol. 2021 Aug 31;12:737474. doi: 10.3389/fphys.2021.737474 (PMC8438321; doi:10.3389/fphys.2021.737474)
Supplement: Supplementary file 1 [file Data_Sheet_1.docx]

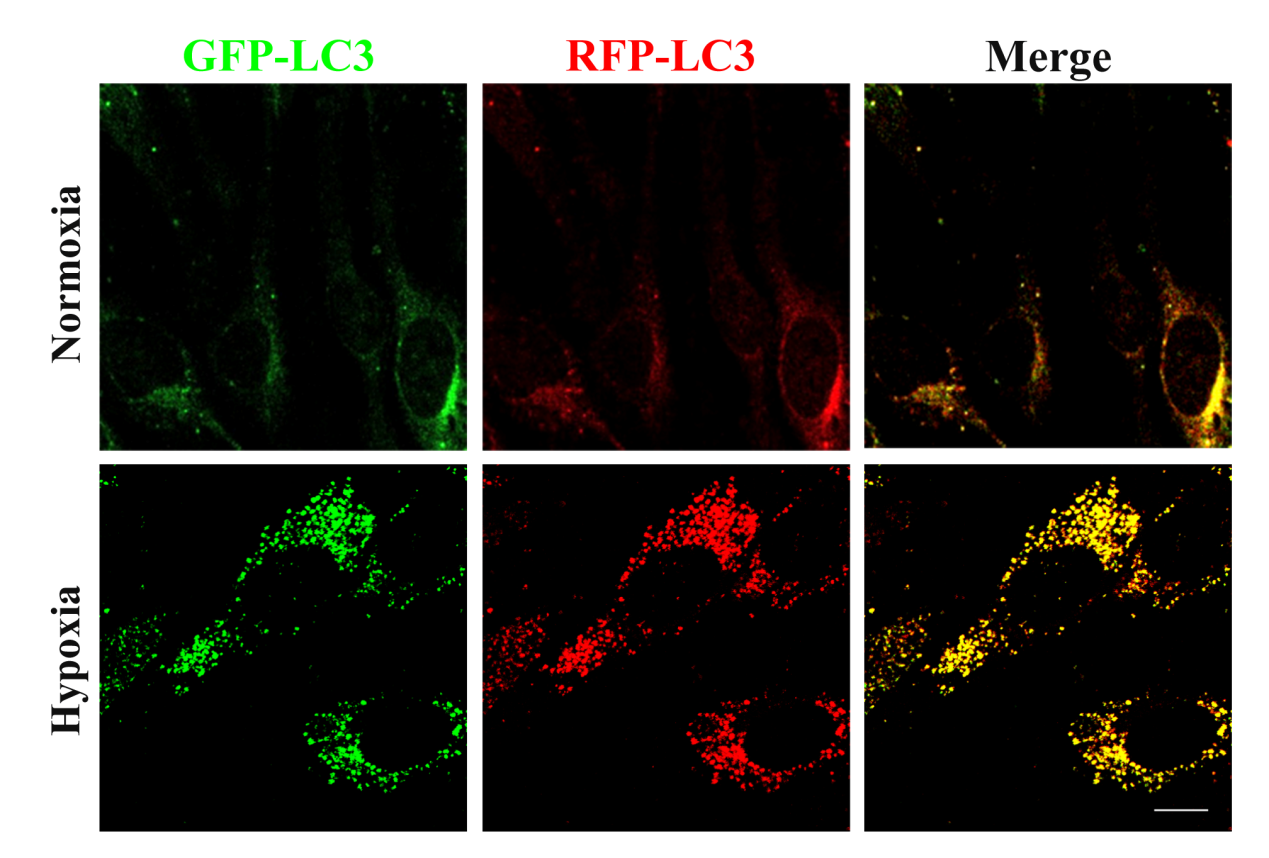


**FIGURE S1** Establishment of a bEnd.3 cell line that stably expresses *RFP-GFP-LC3* adenovirus to determined autophagic flux alterations in bEnd.3 cells in response to hypoxia. Scale bar: 10 μm.


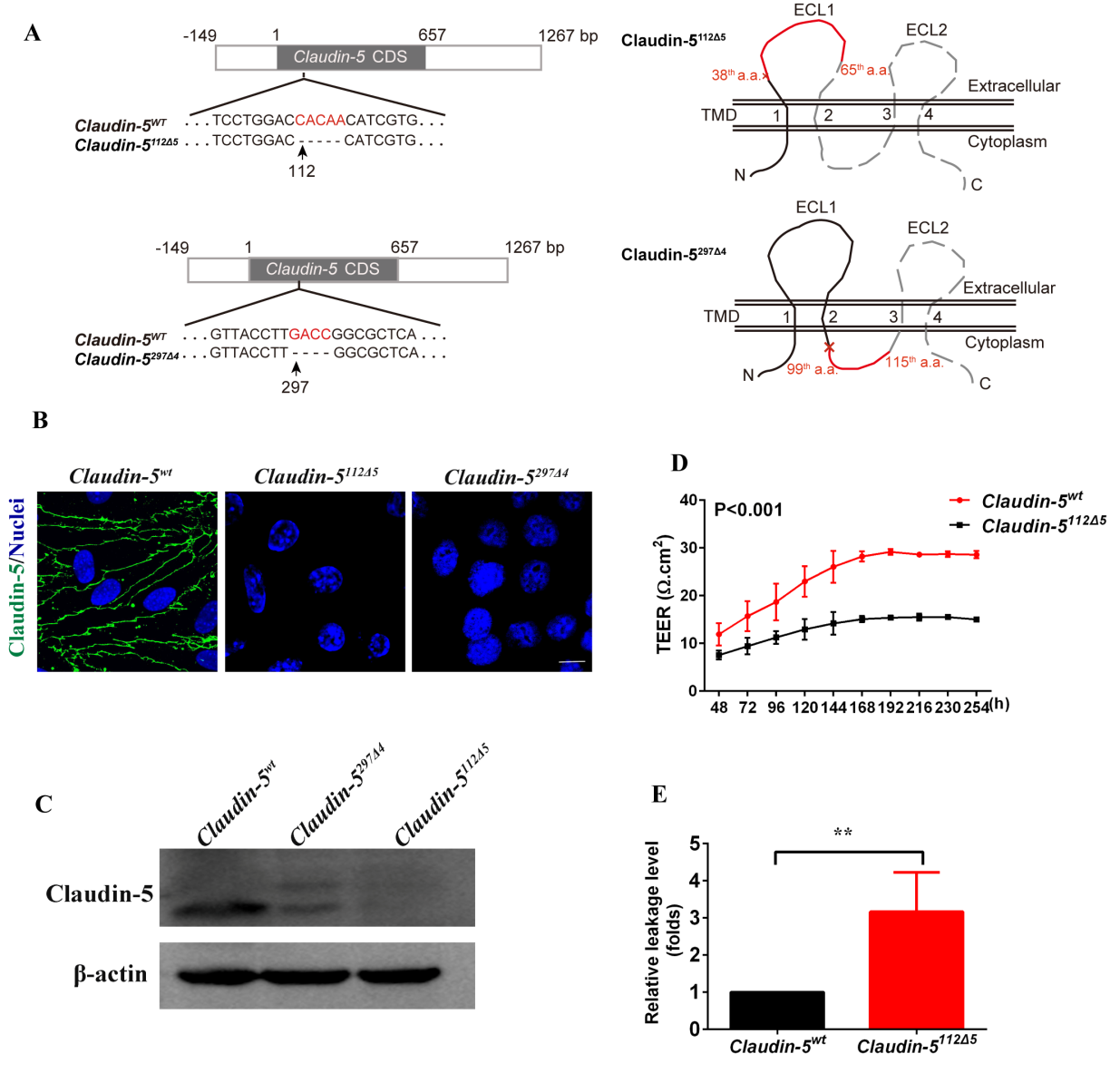


**FIGURE S2** Construction of *Claudin-5* mutant bEnd.3 cell lines and the functional analysis of the monolayer cells. **(A)** Nucleotide sequences of the *Claudin-5* target site. The two *Claudin-5* mutated alleles have a 5 bp deletion (*Claudin-5^112Δ5^*) from nucleotide position 112 and a 4 bp deletion (*Claudin-5^297Δ4^*) from nucleotide position 297 respectively. *Claudin-5^112Δ5^* encodes mutated Claudin-5 from amino acid (a.a.) 38 at the first extracellular loop (ECL1) and a predicted truncation from a.a. 65. *Claudin-5^297Δ4^* encodes mutated Claudin-5 from amino acid a.a. 99 at the intercellular loop and a predicted truncation from a.a. 115. **(B)** Fluorescent immunostaining of Claudin-5 (green) in endothelial cells. **(C)** Western blot analysis of the expression of Claudin-5 in mutate bEnd.3 lines. **(D, E)** Mutant of Claudin-5 in *Claudin-5^112Δ5^* cells causes reduced trans-endothelial electrical resistance (TEER) and increased permeability of monolayer cells in comparison to wild type bEnd.3 cell layer. ******p<0.01. Scale bar, 10 µm. TMD, transmembrane domain.





**FIGURE S3** The activity of the three cell lines in response to hypoxia was analyzed by CCK-8 assays.


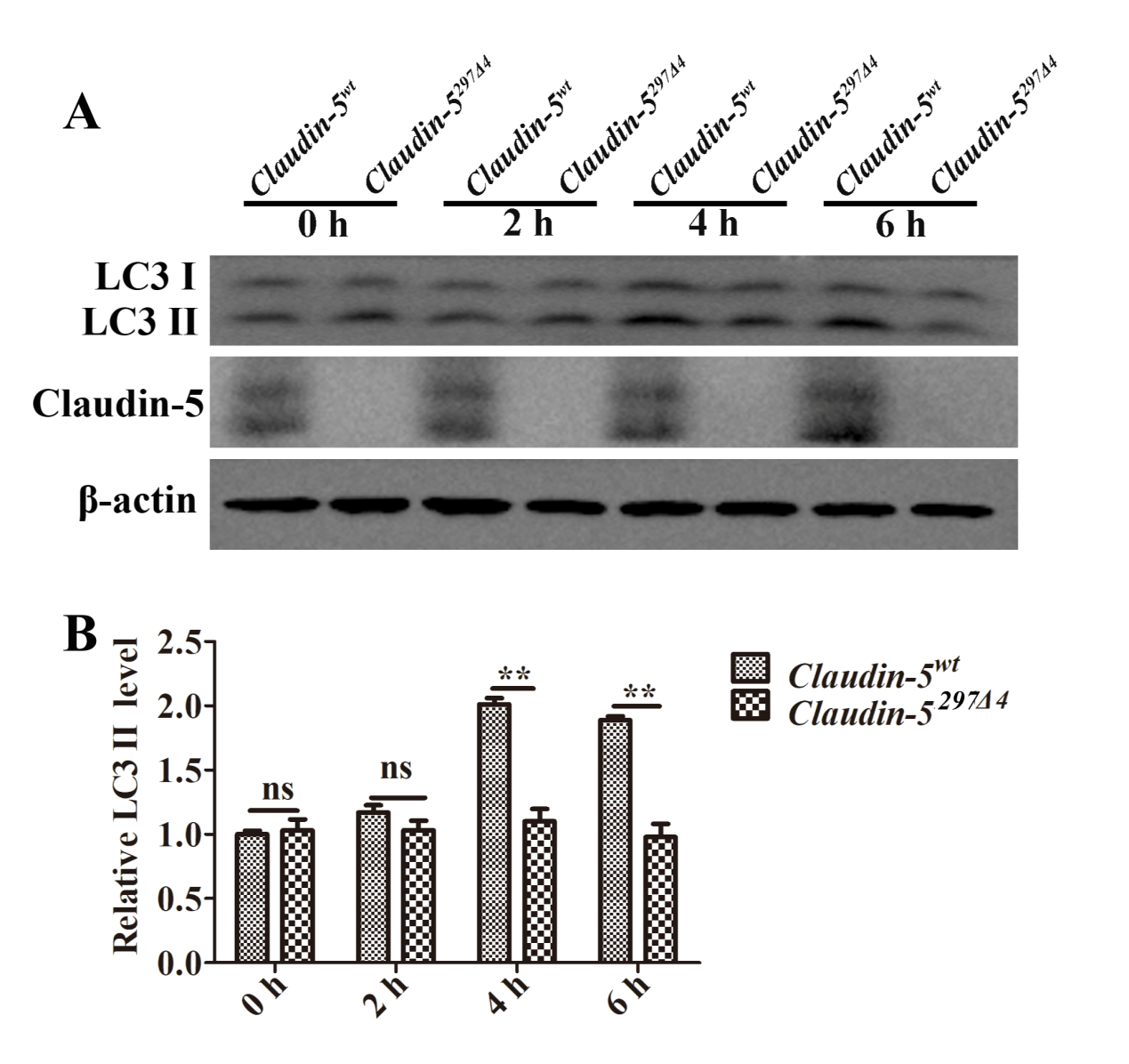


**FIGURE S4** To exclude cell line differences, another mutant cell line, *Claudin-5^297Δ4^* bEnd.3 cell, was analyzed. **(A)** Western blot analysis of LC3. **(B)** Quantitative analysis of LC3 level by ImageJ. Mean ± SD, *n* = 3 independent experiments per group. *****p<0.05, ******p<0.01.


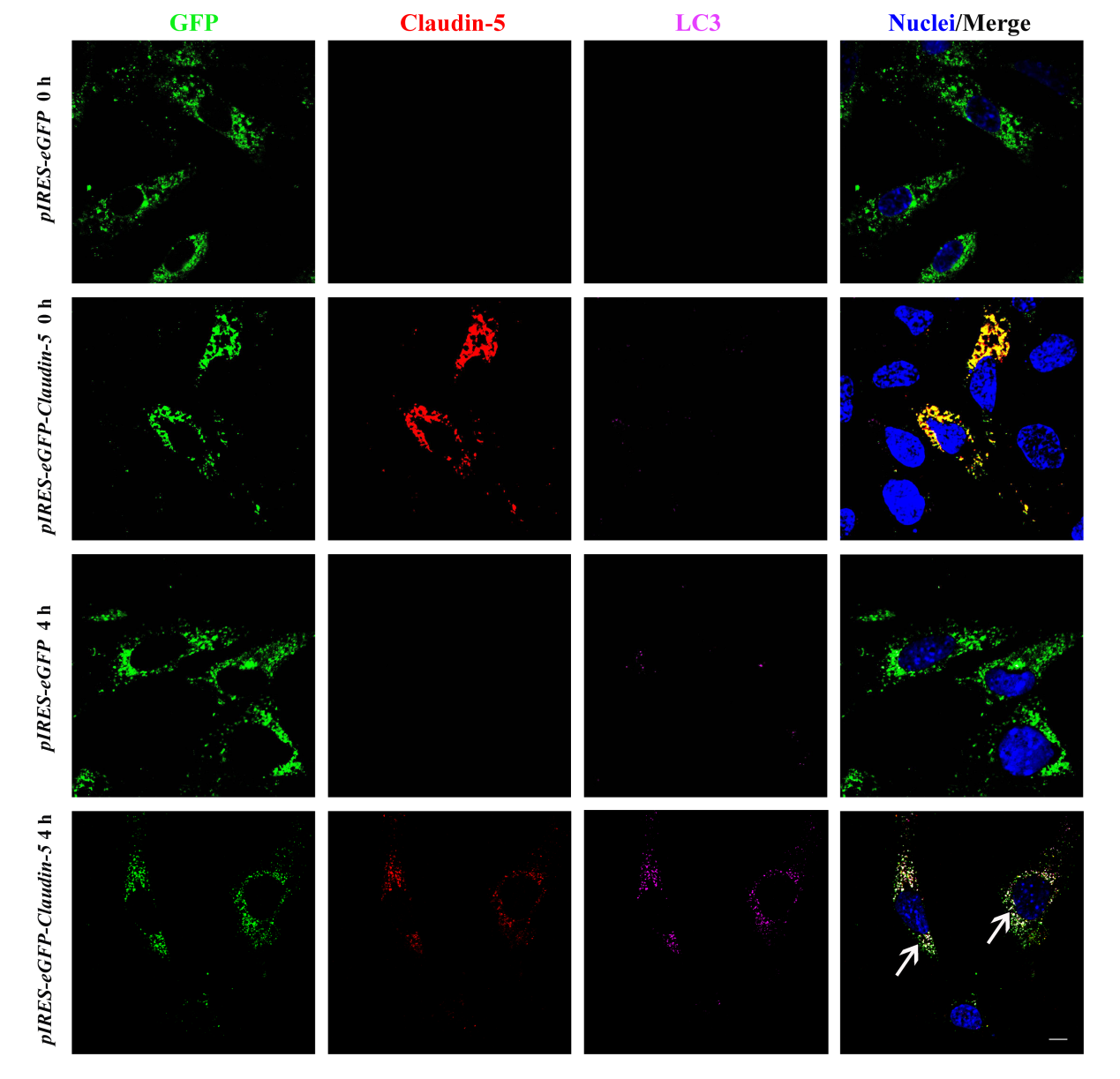


**FIGURE S5** Rescue of *Claudin-5* in *Claudin-5^112Δ5^* bEnd.3 cells; confocal microscopy images of Claudin-5 (red) and LC3 (purple). Scale bar, 10 µm. Mean ± SD, *n* = 3 independent experiments per group.


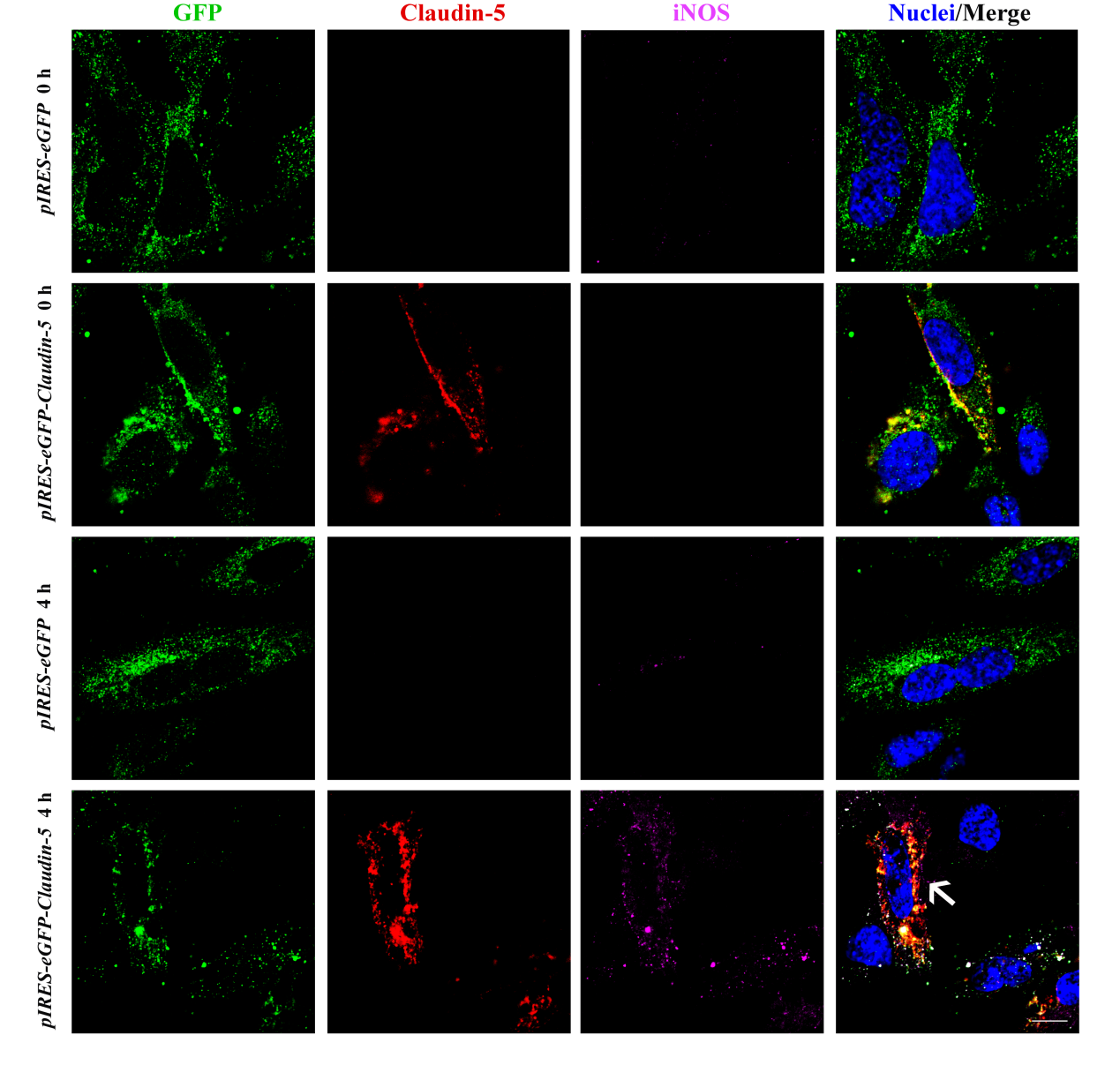
**FIGURE S6** Rescue of *Claudin-5* in *Claudin-5^112Δ5^* bEnd.3 cells; confocal microscopy images of Claudin-5 (red) and iNOS (purple). White arrow indicates the colocalization of Claudin-5 and iNOS. Scale bar, 10 µm. Mean ± SD, *n* = 3 independent experiments per group.


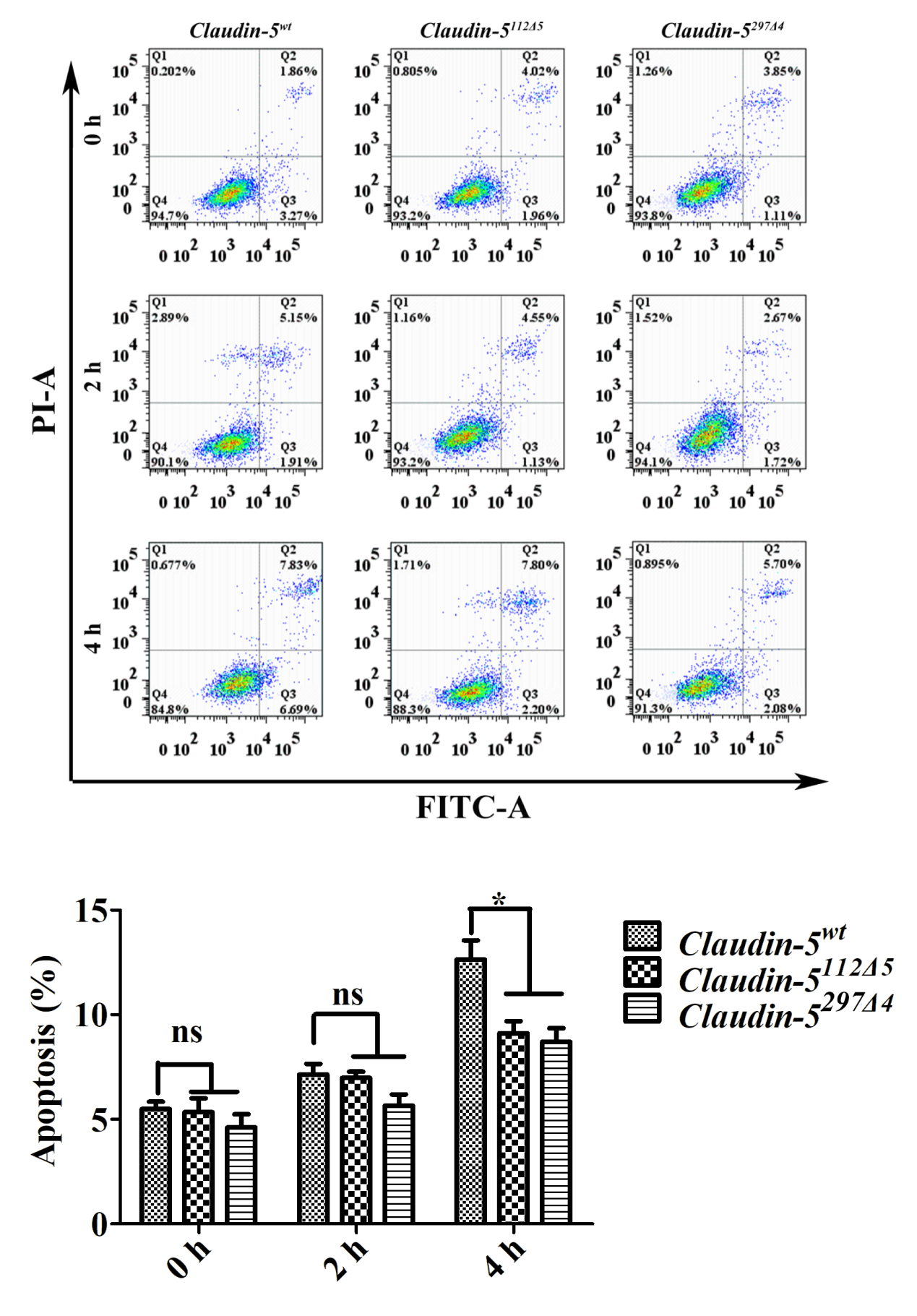


**FIGURE S7** The apoptosis level *Claudin-5* wild type and mutant bEnd.3 cell lines in response to hypoxia was analyzed by flow cytometry. Mean ± SD, *n* = 3 independent experiments per group. *****p<0.05.
